# Reducing Aggression by Developing Emotional and Inhibitory Control

**DOI:** 10.3390/ijerph18105263

**Published:** 2021-05-15

**Authors:** Miriam Romero-López, María Carmen Pichardo, Ana Justicia-Arráez, Judit Bembibre-Serrano

**Affiliations:** Department of Evolutive and Educational Psychology, University of Granada, 18071 Granada, Spain; miriam@ugr.es (M.R.-L.); anajus@ugr.es (A.J.-A.); jbembibre@ugr.es (J.B.-S.)

**Keywords:** emotional control, inhibitory control, self-regulation, aggression, executive functions, teacher training

## Abstract

The objective of this study is to measure the effectiveness of a program on improving inhibitory and emotional control among children. In addition, it is assessed whether the improvement of these skills has an effect on the reduction of aggressive behavior in pre-school children. The participants were 100 children, 50 belonging to the control group and 50 to the experimental group, aged between 5 and 6 years. Pre-intervention and post-intervention measures of inhibitory and emotional control (BRIEF-P) and aggression (BASC) were taken. A Generalized Linear Mixed Model analysis (GLMM) was performed and found that children in the experimental group scored higher on inhibitory and emotional control compared to their peers in the control group. In addition, these improvements have an effect on the decrease in aggressiveness. In conclusion, preventive research should have among its priorities the design of such program given their implications for psychosocial development.

## 1. Introduction

Juvenile delinquency and violence are a public health problem [1]. In Spain, some children and adolescents display aggressive and antisocial behavior, which has been characterized by a significant increase in prevalence in recent years [2]. Aggressive behavior has thus become one of the expensive mental health problems, due to the multiple judicial, social and health care resources it mobilizes [3].

Behavioral problems between the ages of 3 and 5 increase the risk of later exhibiting oppositional defiant disorder, conduct disorder or a depressive episode between the ages of 6 and 13 [4]. In addition, other longitudinal studies indicate that the presence of disruptive behavior in childhood is associated with increased mental health problems, conflicts with the law, substance use, occupational adjustment problems, as well as physical health problems in later life (adolescence and early adulthood) [5,6].

In this line, research shows that it is difficult for a child who has not exhibited any aggression problems before the age of 10 to show behavioral problems thereafter [1,7,8]. Several longitudinal studies show that young people who display severe aggressive behavior in adolescence already exhibited aggressive behavior in childhood [9,10,11]. These results indicate that aggressive behaviors are the result of gradual development, not emerging in isolation in adolescence or adulthood, but beginning in childhood and becoming stronger over the years. 

Therefore, if the chronicity, persistence and occurrence of violent and antisocial behavior and its effects are to be reduced, interventions at the pre-school stage will be necessary. In relation to this stage, the use of physical aggression can be considered normal and adaptive. The first manifestations of physical aggression appear from the age of one year onwards, where they generally manifest themselves through biting, throwing toys and hitting. These behaviors are generally the result of frustration and are reactive in nature, and are not often intended to do harm [12]. Around the age of 2 years, there is an increase in physical aggression until it reaches its peak [13,14]. In this second year, aggressive behaviors are aimed at the acquisition or defense of toys or the intention to achieve a goal [15]. Later, aggression will begin to decline due to the development of inhibitory control and emotional regulation, among others [13,16].

Inhibitory control, one of the basic mechanisms of executive functions, refers to the ability of people to control their emotions, feelings, thoughts and behavior [17,18]. Inhibitory control is vital for adequate social development, as it enables individuals to adjust, adapt and respect established social norms. It also plays an important role both in the establishment of appropriate interpersonal relationships and in the learning process. Inhibitory control is a predictor of adaptive behaviors in childhood [19,20]. However, children with deficits in inhibitory control may experience difficulties in social situations that require cooperative behavior because of their difficulty in inhibiting inappropriate behavior. Thus, such social deficits may predict both internalized and externalized behavioral problems [21]. In fact, many problem behaviors could be conceptualized, in part, as failures to inhibit inappropriate responses [22]. Along these lines, several studies indicate that poor inhibitory control is associated with high levels of aggressive behavior during the pre-school period [14,23,24,25].

Similarly, problems in emotional control are another element that has been considered a risk factor for aggressive behavior [26]. Problems in emotional control refer to the presence of problems in executive functioning within the emotional sphere and consist of difficulty in controlling emotional reactions in different contexts. It is one of the most important intrinsic factors in a child’s overall development and influences academic performance [27,28,29]. Through emotional control, children can control their behavior, thoughts and emotions in the different situations they face in everyday life [30]. Emotional regulation in early childhood education includes students’ use of explicit knowledge about emotions, strategies to modulate and control the expression of emotions, and the ability to get along and resolve conflicts with peers [31,32]. Moreover, emotional regulation is essential because it minimizes the development of problematic behaviors and favors the harmonious and healthy development of people in the context of friendship, love, family, school and work [33]. Likewise, emotional control plays a fundamental role in the prevention and reduction of anxiety, depression and aggressive behavior [34]. Emotions such as fear and anger are associated with aggressive behavior. For this reason, controlling negative emotions can help to reduce aggressive behavior [35,36].

Such findings highlight the importance of designing and implementing programs to improve both emotional and inhibitory control. One of the programs for the improvement of these skills included in the regular curriculum is the program Tools of the Mind: The Vygotskian Approach to Early Childhood Education [37] whose main objective is to enable Early Childhood students to become masters of their own behavior. This includes activities that promote emotional, social and cognitive regulation through attention games, memory games, fantasy games and speech regulation activities. Another of the most widely used programs included in the regular curriculum is the Chicago School Readiness Project (CSRP), whose main objective is to improve the chances of school success of pre-school students through emotional and behavioral training of young children [38]. Other programs, such as the one designed by Traverso, Viterbori and Usai [39] have also been shown to be effective in improving inhibitory control in pre-school pupils. 

However, although most emotional and inhibitory control training programs have been shown to be effective in improving emotional and inhibitory control, it is unclear whether intervention is associated with reduced aggression. Given that studies have shown a relationship between emotional and inhibitory control and aggressive behavior [23,24,25,33,34,35,36,40,41] one might expect that improvement in these skills would be linked to a reduction in aggressive behavior. However, studies show that learners have difficulties in transferring what they have learnt in one task to another untrained task [42,43]. In this line, studies on inhibitory control have found that the effects of inhibitory control on other untrained variables are minimal. The study by Thorell et al. demonstrated the difficulty preschool children have in transferring improvements in inhibition to other untrained tasks. In this study, it was found that, although inhibitory control training improved the trained tasks, these improvements did not transfer to other variables such as attention or working memory [44].

In relation to emotional control, there are no known studies whose objective is to analyze whether the improvement of this variable has an impact on the reduction of aggressive behavior. However, research along these lines is needed, as the long-term goal of emotional and cognitive training is to improve people’s daily functioning [45]. Therefore, if training in inhibitory and emotional control is to be effective in reducing aggressiveness, it is necessary to include activities that favor generalization, including activities in the programs that favor positive student relationships based on emotional and inhibitory control.

In addition, designing programs within the regular curriculum that pre-school teachers can apply as a methodological resource in the classroom can have positive effects on improving the social and cognitive functioning of pupils. At the pre-school stage, these benefits are even greater, as some research has found that children at this stage of education benefit more from intervention programs than pupils at later stages of education [46].

Thus, based on the theoretical review carried out, the objectives and contributions of this study are as follows:

1. To expose the effects of a training program in inhibitory and emotional control, designed for the present research and whose main objective is the improvement of these components, focusing not only on cognitive processes but also on the more behavioral part associated with these capacities. As far as we know, there are no programs that work on inhibitory and emotional control that can be applied by teachers as part of the regular curriculum in the pre-school stage and that have been validated in the Spanish population. The program is designed to be implemented by teachers after their training. Teacher training equips pre-school teachers with strategies and tools for the proper development of their students.
2. The second objective is to analyze whether the improvement of inhibitory and emotional control has an effect on the reduction of aggressive behavior in pre-school pupils. In this case, there are studies with contradictory or inconsistent results that need to be clarified. Kassai, Futo, Demetrovics and Takacs [43] conducted a meta-analysis where they found that pre-school students have difficulties in transferring the skills acquired through executive coaching to other activities of daily life. However, Scionti, Cavallero, Zogmaister and Marzocchi [47] conducted another meta-analysis where they found transfer effects. However, there are no known studies that examine whether emotional and inhibitory control has effects on other social domains such as aggression.

Since the program created for this research has been designed to include social activities where executive functions play a key role, it is expected that children participating in the program will not only gain improvements in inhibitory and emotional control, but will also reduce their aggressive behaviors compared to their peers in the control group. 

## 2. Materials and Methods

### 2.1. Participants

The participants are 100 pupils, aged 5–6 years (Mage = 5.48, SDage = 0.23), enrolled in the last year of pre-school education. The control group consists of 50 children, 23 girls and 27 boys, as well as the experimental group which also consists of 50 pupils, 23 girls and 27 boys. Children were excluded if they had an intellectual disability, sensory-motor disability, chronic illness or neurodevelopmental disorder. For this reason, although the initial number of participants was 110 pupils, 10 children were excluded because they did not meet the requirements.

All participants attend the same school. This decision was taken in order to ensure that all pupils receive the same educational methods, to control the physical spaces, the philosophy of the school and the socio-demographic variables of the parents, since in Spain the main criterion for the allocation of schools is the proximity of the school to the place of residence. All students were Caucasian and European with a middle socio-economic status.

### 2.2. Instrument

Inhibitory and emotional control were assessed through the Behavioral Rating Inventory of Executive Function-Preschool Version (BRIEF-P) [48,49]. It is a Likert-type scale with 63 items and three response possibilities (never, sometimes and often). However, only the 26 items corresponding to the following scales were used for this research: deficits in inhibition (α = 0.96), evaluating the presence of inhibitory control problems in the child—that is, in the capacity to inhibit, resist or not react to an impulse—as well as the existence of difficulties in halting or recognizing behavior at the appropriate moment (16 items, e.g., “Is unaware of how his/her behavior affects or bothers others”) and deficits in emotional control (α = 0.89), establishing whether the child manifests problems with executive function within the emotional sphere, and evaluating the existence of difficulties in moderating emotional responses (10 items, e.g., “Overreacts to small problems”).

On the other hand, the aggressiveness scale of the Behavioral Assessment System for Children and Adolescents (BASC) questionnaire was used to assess aggressive behavior [50,51]. This scale is composed of 5 global dimensions and 14 clinical scales. However, only the 12 items corresponding to the aggression scale, which assesses the child’s tendency to act in a hostile way, either verbally or physically threatening others (e.g., “hitting other children”), were used for the present research. The internal consistency index obtained in the present research on the aggressiveness scale is α = 0.92.

### 2.3. Inhibitory Control and Emotional Control Training Program

The program for the improvement of inhibitory and emotional control has been designed for children aged 5 to 6 years. It consists of 16 sessions of approximately 30 min that are applied twice a week. It includes methodologies such as case studies, problem-based learning, cooperative enquiry and simulation games. All sessions use games in order to motivate students. In addition, it takes into account all the more behavioral aspects of these executive functions necessary for proper social interaction. In this way, it includes both cognitive and behavioral activities. The 16 sessions begin with a social dilemma where both inhibitory and emotional control play a key role. For example, Carla and Pepe, the protagonists of the program, are presented fighting because they both want to be first in line. When she does not succeed, Carla hits Pepe in order to achieve her goal. The students then discuss how the protagonists could have resolved the conflict in a more positive way and how they think the two characters felt. Students then reflect on similar situations they have experienced and how they have felt in these situations. In addition, they are taught different impulse and emotional control techniques, such as the traffic light technique or the turtle technique, and role-plays are performed to guide them through various solutions they should have applied.

In addition, the program includes visual, plastic, auditory and physical activities. For example, the emotional die is included, in which children roll a die with different emotions (sadness, anger, rage, joy, calm and fear). Once they have rolled the dice, they should draw a situation in which they have felt that emotion. In addition, when the emotion of anger or rage comes up, they will reflect with them on how to control these negative emotions and how to feel calm through the use of different techniques such as relaxation, the turtle or the traffic light.

Similarly, the program also includes tasks that are used for the assessment of inhibitory control, such as the Stroop task in which students are shown pictures with opposite pictures (e.g., night-day, happy-sad, …) and have to say the opposite word to the picture shown, e.g., “night” when shown a picture of sunrise and “day” when shown a picture of sunset.

### 2.4. Procedure

Before starting the research, a project was drawn up in which all the objectives and requirements necessary to carry out the research were set out. The project was then sent to the Bioethics Committee on Human Research of the University of Granada to ensure that it complied with all the requirements of the Code of Ethics in Psychology and the data protection law. 

Once the project was approved, a survey was carried out to find out whether the directors of all the pre-schools in the capital of Granada were interested in implementing the program and in teacher training. Several schools confirmed their interest in participating in the research and among them a random selection was made and one school was selected.

Once the school where the research was carried out had been selected, a meeting was held with all the families of the pre-school pupils. The purpose of this meeting was to explain the objectives of the research and for families interested in their children’s participation to sign the informed consent form. Families who could not attend the meeting were sent a letter compiling all the information discussed at the meeting and those families interested in their children’s participation returned the signed letters.

Subsequently, a person was recruited and trained to carry out the evaluations of the participating students. For this purpose, during the observation phase (pre and post), the evaluator was given a record sheet with the behaviors to be analyzed and was asked to indicate their frequency both in the classroom and in the playground. Subsequently, on the basis of this registration sheet, he was asked to fill in the standardized questionnaires used in the research. During these two months, a pre-school teacher, who was hired for the research, was also trained to implement the inhibitory and emotional control improvement program and was told which activities to implement with the active control group. Both the evaluator and the teacher in charge of implementing the interventions were unaware of the objectives of the research, using a double-blind procedure (intervention and evaluation).

The person hired to carry out the evaluation observed the behavior of both the experimental group and the active control group for 4 h a day for two months. For this purpose, it was taken into consideration that the observation was carried out on similar days, places and at similar times in both groups. After two months, the evaluator completed the scales of inhibitory and emotional control (through the BRIEF-P) and the aggressiveness scale (BASC) for each of the students who participated in the study (pre-intervention phase).

When the evaluator had completed all the scales, the intervention phase began, which lasted two and a half months and was carried out by the early childhood teacher hired for this research. Thus, he carried out different activities in the active control group and applied the emotional and inhibitory control improvement program in the experimental group. Activities in the control group included construction games, storytelling or drawing a picture. As with the evaluation, the days and times were rotated so that both groups (experimental and control) had similar schedules.

The educational center where the research was carried out provided a classroom usually used by the students for sports activities, to carry out the tasks designed for both the experimental group and the active control group. The teacher contracted for the implementation of the program took the students assigned to each group to the classroom on the scheduled days and times. The rest of the students remained in regular classes with their teacher. 

One month after the intervention, the post-intervention evaluation phase began. The same evaluator from the pre-intervention phase again observed the behavior of the pupils both in the classroom and in the playground for two months. After this period of time, the evaluator completed the inhibitory and emotional control scales (via the BRIEF-P) and the aggression scale (BASC) for each participating child (post-intervention phase).

**Figure 1:**
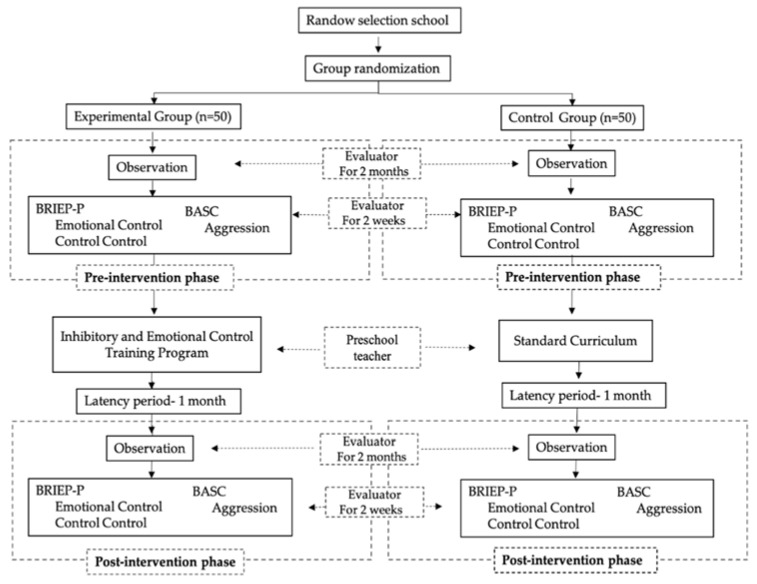
Time distribution of study activities.

Figure 1 shows the activities that have been carried out in this research, as well as the tasks performed and the responsible personnel.

Afterwards, data analysis was carried out and the research report was written. Finally, the school was informed of the results obtained.

### 2.5. Design and Statistical Analysis

Taking into account the objectives of the study, an individual randomized trial experimental study was designed with two groups (experimental and control) and two evaluation phases (pre-intervention and post-intervention). 

Initially, descriptive analyses are carried out in which the means and standard deviations of the variables considered are presented. Given the distribution of the data, and that this was a repeated measures study, Generalized Linear Mixed Model (GLMM) analysis with logit link function with Poisson distribution with a random intercept for each subject was used to compare the two groups on the primary outcomes at two time points. The independent variables in the model included a binary variable for group assignment (intervention vs. control), a binary variable for time (pre-intervention vs. post-intervention) and their interaction term. Comparisons were made between the control and intervention groups in the two phases considered (Pre-intervention and Post-intervention) using a t test with sequential Bonferroni adjustment. Finally, the effect size of the differences between the control and experimental groups is included. Taking into account that the distribution of the data analyzed for the different variables did not have a normal distribution, a non-parametric mean difference test (Mann–Whitney U) was performed to obtain the effect size of the intervention. Finally, Cohen’s d was performed, using the non-parametric test values [52]. Cohen established large (d ≥ 0.80), medium (0.50 ≤ d ≤ 0.79) and small (0.20 ≤ d ≤ 0.49) effects [53].

The Statistics 24 for Mac version of the Statistical Package for the Social Sciences (SPSS) (IBM, Armonk, NY, USA) was used for the different analyses carried out in.

## 3. Results

Table 1 shows the descriptive data for each of the variables analyzed in the control and experimental groups in the pre-intervention and post-intervention phases.

As can be seen in Table 2, the preliminary comparative fixed effects analysis on the three variables analyzed was significant when comparing Inhibitory Control (*F*(1, 196) = 15.97; *p* < 0.001), Emotional Control (*F*(1, 196) = 18.98; *p* < 0.001) and Aggression (*F*(1, 196) = 52.05; *p* < 0.001) by group. Significant differences were also observed in the comparison between the pre-intervention and post-intervention phases in Inhibitory Control (*F*(1, 196) = 127.42; *p* < 0.001), Emotional Control (*F*(1, 196) = 71.06; *p* < 0.001) and Aggression (*F*(1, 196) = 123.59; *p* < 0.001). Group* Time interaction effects are also found for Inhibitory Control (*F*(1, 196) = 90.50; *p* < 0.001), Emotional Control (*F*(1, 196) = 41.86; *p* < 0.001) and Aggression (*F*(1, 196) = 83.40; *p* < 0.001).

On the other hand, being repeated measures, the predictive analysis taking into account as random effects the scores of each participant in each phase, differences are observed between the experimental and control groups in Inhibitory Control, Emotional Control and Aggression. In terms of the differences between intervention times, no significant differences were observed in the variables Inhibitory Control and Emotional Control. On the other hand, there are differences in the variable Aggression (*t*(196) = 2.36; *p* < 0.019). The Group*Time interactions were significant in all the variables analyzed. 

When the model performs the pairwise contrast with the estimated means, as shown in Table 3, in the Inhibitory Control problems there are differences between the experimental and control groups, with the control group participants obtaining higher scores (*t*(196) = −3.62; *p* < 0.001). Differences were also observed in this variable between pre-intervention and post-intervention times, with higher scores in the first phase (*t*(196) = 7.78; *p* < 0.001). In Emotional Control problems, no significant differences were observed between groups or between the pre-intervention and post-intervention phases. On the other hand, in Aggression problems there are significant differences between groups (*t*(196) = −5.46; *p* < 0.001), with higher scores in the control group. Similarly, differences were also observed between the pre- and post-intervention phases (*t*(196) = 7.83; *p* < 0.001), with higher scores in the pre-intervention phase.

Table 4 shows the pairwise contrasts for the interaction Group*Time, taking the estimated means of the model as a reference. The results obtained show significant differences in the experimental group between the pre-intervention and post-intervention phases in the three variables analyzed. Participants in the experimental group scored lower on Inhibitory Control (*t*(196) = 6.29; *p* < 0.001), Emotional Control (*t*(196) = 6.16; *p* < 0.001) and Aggression (*t*(196) = 6.02; *p* < 0.001) problems after participating in the intervention program.

In the control group, no significant differences were observed between phases in Inhibitory Control and Emotional Control, but a significant reduction in Aggression levels was observed in the post-intervention phase (*t*(196) = 2.25; *p* < 0.026). However, the estimate of change is lower than that observed in the experimental group.

Finally, the effect sizes in the comparison between the control and experimental groups, performed from the non-parametric Mann–Whitney test, show high effect sizes in all comparisons: Inhibitory Control (*d* = 1.5); Emotional Control (*d* = 1.3); and Aggression (*d* = 2.1).

## 4. Discussion

The objectives of this study were to determine the effectiveness of a training program included in the regular curriculum for the improvement of inhibitory and emotional control and to analyze whether the improvement of these components has an effect on the reduction of aggressive behavior in pre-school pupils.

Firstly, the effectiveness of the program in improving inhibitory and emotional control was examined. The results of the study confirm the first hypothesis since the students who participated in the program obtained higher scores in inhibitory control and emotional control than their peers in the active control group. The experimental group exhibited a greater ability than the active control group to resist, inhibit and not react to their impulses and were able to withhold inappropriate behaviors when the situation called for it. In addition, the experimental group had an easier time regulating their emotional responses than the active control group. Therefore, the effectiveness of the program is confirmed as children who participated in the inhibitory and emotional control improvement program improved their scores on these variables compared to their peers who only received standard curricular activities.

In line with the results of this research, other studies have also shown the effectiveness of executive function training programs. For example, in the study by Traverso et al., involving 75 five-year-old children (43 controls and 32 experimental), they demonstrated the effectiveness of an intervention program on executive functions with 12 30-min sessions carried out during one month. Children in the experimental group scored higher on inhibition, working memory and cognitive flexibility than their peers in the control group. However, these authors did not include emotional control as a variable in their study [38]. Similarly, the study by Diamond, Lee, Senften, Lam and Abbott found that the Tools of the Mind program increased the daily executive abilities of participating students compared to their peers in the control group [18].

In relation to the improvement of emotional control in pre-school education, Graziano and Hart carried out a study with 45 pre-school children with externalizing behavior problems in which they applied three programs: one for school readiness, another program that also included behavior modification guidelines and a third program that included activities for the improvement of emotional self-regulation. The results showed that students participating in all programs significantly improved their behavioral functioning by a similar magnitude. However, children who participated in the emotional self-regulation program experienced greater growth over time in emotion knowledge, academic performance, executive functioning and emotion regulation compared to children in the other groups. However, in this study they focused only on children with externalizing behavioral problems. While it is vital to design such programs for the population with externalizing behavioral problems, it is also necessary to design programs that aim not only to intervene when problems already exist, but also to prevent these behaviors [26].

The second hypothesis was also confirmed, as students who participated in the program reduced their aggressive behavior compared to their peers in the active control group. Thus, children in the control group acted more hostile, both verbally and physically, compared to their peers who participated in the program. These results may mean that children in the experimental group became more self-regulated and learned to solve interpersonal problems more effectively, and this improvement seems to be related to the impact of the program on emotional regulation and control of inappropriate behavior.

Similarly, another study by Dias, Trevisan, Leon, Prust, and Seabra involving 180 preschool children found that executive functions played an important role in explanatory models of behavior, playing a key role in the prevention of behavioral problems [40]. These results are also supported by the study of Romero, Pichardo, Ingoglia, and Justicia in which 260 5-year-old and 6-year-old students participated. These authors found that all components of executive functions predicted at least one factor of conduct problems, concluding that executive functions act as a protective factor for conduct problems and are important in the development of social competence [41].

Along these lines, another study by Dias and Seabra found similar results. These authors carried out a study involving 58 children (30 belonging to the control group and 28 to the experimental group) aged between 3 and 6 years in which they applied a program for the improvement of executive functions. They found that children in the experimental group improved their executive functions compared to children in the control group and that these improvements had an effect on reducing behavioral problems [54].

The program applied in the study by Dias and Seabra has in common with the one used in this research, that both programs include activities in which executive functions are used to successfully cope with the demands of daily life in children’s contexts.

However, not all studies have found these results. Kassai, Futo, Demetrovics and Takacs conducted a meta-analysis with the aim of finding out whether the improvement in executive functions had far and near transfer effects. These authors defined near transfer as the improvement of a different executive component than the one being trained, for example, if the improvement in inhibition is transferred to the improvement in cognitive flexibility. However, distant transfer was defined as transfer to other skills not directly trained, such as aggression. These authors found that, although the children improved their executive functions, these improvements did not transfer to other untrained domains [43].

The presence of contradictory results could be explained by the activities included in the programs. Children improve those skills they practice, but have difficulty transferring these improvements to other contexts or very different activities. Therefore, it is important to include real-world activities in executive coaching programs, as the aim of any executive intervention should be to improve the impact of these skills in the day-to-day life of the learner. Programs that include real-world, curriculum-based activities have been shown to be more effective than computer-based or purely cognitive programs [42].

Therefore, for inhibitory and emotional control training to be effective in reducing aggression, it is necessary to include activities where these skills are necessary for the establishment of satisfactory social relationships.

On the other hand, the children in the control group, despite not participating in the program, also decreased their aggressive behavior in the post-program phase, although not to the same extent as the children in the experimental group. These results also seem logical since pre-school children are in the midst of social development. Furthermore, school is one of the most important socializing agents in the infant stage and the simple fact of attending an educational center could encourage behavioral control and respect for the rules of coexistence. These results are congruent with those found in other studies such as the one carried out by Alba, Fernández-Cabezas, Justicia, and Pichardo who found that after the application of a program to improve social competence, both the students in the control group and the experimental group obtained lower scores in behavioral problems. For this reason, it could be concluded that the education received in pre-schools could favor the development of self-regulation and the reduction of behavioral problems such as aggression [55].

Taking into account the results found, it is concluded that intervention in inhibitory and emotional control can be useful to accelerate the improvement of these skills in pre-school education and that these improvements can favor the reduction of aggressive behavior. However, it stresses the importance of including in intervention programs concrete activities that favor this transfer.

However, the present research has a number of limitations that need to be taken into account before extrapolating the results. The first is that the study was carried out only in a pre-school in the province of Granada (Spain), which makes external validity difficult, i.e., extrapolating these results to other contexts. For this reason, it would be interesting to apply the program in other schools in other areas and to widen the age range in which it is applied.

The next limitation of the study is related to the evaluation. As mentioned, the evaluation was carried out by a person contracted for the present research. This decision was made in order to ensure objectivity. However, it would be interesting to contrast this information with that provided by other people close to the students, such as families or teachers, in order to favor hetero-evaluation.

Finally, it would also be necessary to analyze the long-term impact of the program in a third follow-up phase and to include other variables that may be crucial in cognitive and social development, such as parenting practices employed by families.

Despite these limitations, the inhibitory and emotional control enhancement program used in this research has a number of strengths that are also important to mention. Firstly, the usefulness for teachers, as it enables them to have at their disposal a series of activities that not only improve inhibitory and emotional control, but also reduce aggressiveness. This research shows that emotional and inhibitory control can be promoted using a program that can be applied in the classroom, in regular classes and that can be easily applied by teachers. In addition, this program has other advantages such as the possibility of reaching a larger number of children (compared to other programs applied in a clinical setting) and its potential to prevent aggressive behavior. In addition, it is a program that uses inexpensive materials, which means that it can be implemented in any school even if the school is low-income. In this way, it could be applied in schools located in at-risk contexts, where students generally present greater problems in emotional and cognitive self-regulation and more aggressive behavior, with the aim of reducing the gap in the level of executive functioning in at-risk students.

Therefore, the design of executive education programs should be considered a priority for preventive and applied research in the fields of psychosocial development.

## 5. Conclusions

In conclusion, the program for the improvement of emotional and inhibitory control designed for this research could be an effective tool for the improvement of these skills in the infant stage. Furthermore, the study showed that improving these skills has an effect on reducing aggressive behavior.

## Figures and Tables

**Table 1:** Descriptive date in Inhibitory Control, Emotional Control and Aggression by groups and phases.

Outcome Variable	Pre-Intervention			Post-Intervention		
	M (SD)	Min.	Max.	M (SD)	Min.	Max.
G. Experimental						
Inhibitory Control	6.36 (0.85)	0.00	23.00	1.18 (0.26)	0.00	7.00
Emotional Control	3.60 (0.49)	0.00	12.00	0.78 (0.20)	0.00	6.00
Aggression	5.72 (0.89)	0.00	21.00	1.12 (0.24)	0.00	7.00
G. Control						
Inhibitory Control	7.02 (1.01)	0.00	26.00	6.08 (0.87)	0.00	23.00
Emotional Control	4.06 (0.39)	0.00	10.00	3.32 (0.37)	0.00	10.00
Aggression	9.48 (0.80)	1.00	22.00	8.08 (0.73)	0.00	21.00

Note. Inhibitory Control = deficit in inhibitory control; Emotional Control = deficits in emotional control.

**Table 2:** Results of Generalized Linear Mixed Models estimating change in outcomes.

Outcome Variable Parameter	*F*	Coefficient (SE)	*t*	*p*-Value	C.I 95%	
					Inferior	Superior
Inhibitory Control						
Group	15.97 ***	−1.62 (0.24)	−6.67	<0.001	−2.10	−1.14
Time	127.42 ***	0.14 (0.08)	1.83	0.068	−0.01	0.30
Group*Time	90.50 ***	1.54 (0.16)	9.51	<0.001	1.22	1.86
Emotional Control						
Group	18.98 ***	−1.54 (0.25)	−6.21	<0.001	−2.02	−1.05
Time	71.06 ***	0.20 (0.10)	1.92	0.056	−0.01	0.41
Group*Time	41.86 ***	1.33 (0.20)	6.47	<0.001	0.92	1.73
Aggression						
Group	52.05 ***	−2.28 (0.24)	−9.33	<0.001	−2.77	−1.80
Time	123.59 ***	0.16 (0.07)	2.36	0.019	0.03	0.29
Group*Time	83.40 ***	1.47 (0.16)	9.13	<0.001	1.15	1.79

Note. Inhibitory Control = deficit in inhibitory control; Emotional Control = deficits in emotional control. SE = Standard error. C.I = Confidence Intervals. *** *p* < 0.001.

**Table 3:** Pairwise contrasts of Groups and Times, using the means estimated by the model.

Outcome Variable Parameter	Estimation (SD)	*t*	*p*-Value	C.I 95%	
				Inferior	Superior
Inhibitory Control					
Group (Exp-Cont.)	−2.58 (0.71)	−3.62	<0.001	−3.98	−1.17
Time (pre-pos)	2.78 (0.35)	7.88	<0.001	2.09	3.48
Emotional Control					
Group (Exp-Cont.)	−2.22 (3.42)	−0.65	0.516	−8.96	4.52
Time (pre-pos)	2.06 (2.36)	0.87	0.384	−2.59	6.72
Aggression					
Group (Exp-Cont.)	−5.70 (0.91)	−5.46	<0.001	−7.76	−3.64
Time (pre-pos)	2.09 (0.39)	7.83	<0.001	2.31	3.87

Note. Inhibitory Control = deficit in inhibitory control; Emotional Control = deficits in emotional control. SD = Standard deviation. C.I = Confidence Intervals.

**Table 4:** Pairwise contrasts in the Group*Time interaction using the model-estimated means.

Outcome Variable Parameter	Estimation (SD)	*t*	*p*-Value	C.I 95%	
				Inferior	Superior
Inhibitory Control					
Experimental (pre-pos)	3.63 (0.58)	6.29	<0.001	2.50	4.78
Control (pre-pos)	0.65 (0.36)	1.78	0.077	−0.07	1.36
Emotional Control					
Experimental (pre-pos)	2.14 (0.39)	6.16	<0.001	1.46	2.83
Control (pre-pos)	0.61 (0.33)	1.88	0.062	−0.03	1.26
Aggression					
Experimental (pre-pos)	2.80 (0.46)	6.02	<0.001	1.88	3.71
Control (pre-pos)	1.16 (0.51)	2.25	0.026	−2.17	0.14

Note. Inhibitory Control = deficit in inhibitory control; Emotional Control = deficits in emotional control. SD = Standard deviation C.I = Confidence Intervals.

## Data Availability

Data available on request due to ethical restrictions.
